# Yew (*Taxus*) intoxication in free-ranging cervids

**DOI:** 10.1371/journal.pone.0188961

**Published:** 2017-12-27

**Authors:** Kjell Handeland, Turid Vikøren, Terje D. Josefsen, Knut Madslien, Belinda Valdecanas, Silvio Uhlig

**Affiliations:** 1 Norwegian Veterinary Institute, Oslo, Norway; 2 Norwegian Veterinary Institute, Tromsø, Norway; University of Missouri Columbia, UNITED STATES

## Abstract

Wild ruminants, including deer species (cervids) have incorrectly been regarded as refractory to yew (*Taxus*) intoxication. This assumption has been based upon anecdotal observations of individual deer browsing on yew over time without apparent adverse effect. A single case of yew intoxication was reported in a free-ranging Norwegian moose (*Alces alces*) in 2008. The current report describes five additional cases of yew toxicosis in moose, seven in roe deer (*Capreolus capreolus*) and two in reindeer (*Rangifer tarandus tarandus*), all in Norway. The animals were found dead during the winter, close to or within gardens containing yew plants showing signs of browsing. Gross findings included lung congestion and edema, thoracic and pericardial effusion, bilateral heart dilatation, epi- and endocardial hemorrhage, and enlarged (congested) spleen. Yew plant remnants were detected in the rumen of all animals with the exception of a single moose. Histology revealed multifocal acute myocardial degeneration and necrosis with hemorrhage in roe deer, but not in the two other species. A qualitative high performance liquid chromatography–ion trap mass spectrometry analysis was used to tentatively identify five major *Taxus* alkaloids (taxines) in crude yew extracts and in heart and liver samples from the moose cases. All five major taxines were detected with good signal/noise ratio in tissue samples from the four moose with visible ruminal yew content, whereas lower levels of taxines were detected in the moose without visible ruminal yew content. Possible differences in interspecies tolerance to taxines and role of individual protective adaptation are discussed.

## Introduction

Yew (*Taxus* spp.) poisoning has been recognized in livestock and humans for hundreds of years, whereas free-ranging ruminants like deer species (cervids) have been regarded as tolerant [1]. This assumption has been based upon observation of individual deer e.g. European roe deer (*Capreolus capreolus*) or North American white-tailed deer (*Odocoileus virginianus*), browsing yew over time without apparent adverse effect [2–4]. Cases of acute intoxication have, however, been reported in captive fallow deer (*Dama dama*) fed clippings from ornamental yew [5], [6]. An important question is whether the supposed tolerance among free-ranging deer may reflect individual adaptation following induction of protective detoxification mechanisms e.g. cytochrome P-450 enzymes, in the liver. In ruminants, also microbial degradation of taxines in the rumen is considered important [7]. The first case of yew intoxication in a free-ranging ruminant was described in moose (*Alces alces*) in Norway in 2008 [8]. In January 2017, yew-induced mass mortality in a flock of free-ranging pronghorn antelopes (*Antilocapra americana*) was a reported by Idaho Fish and Game, USA (http://promedmail.org>outbreakwatch.blogspot.com>2017/01). Yew intoxication of eight elk (*Cervus canadensis*) was also reported in the same report.

Yew plants contain a heterogeneous mixture of cardiotoxic alkaloids; the major constituents being taxine A and taxine B [9]. Taxine B and its derivatives constitute the main and most toxic group. Taxines act as calcium and sodium channel antagonists within cardiac myocytes and may induce cardiac arrhythmia, atrioventricular block and diastolic cardiac arrest [10]. Death through acute heart failure normally occurs in less than 24 hours. However, in cattle a subacute to chronic intoxication lasting up to 18 days has been reported [11–15]. In such cases, detection of yew remnants in the rumen may be difficult. Detection of yew remnants within the rumen of moose can be considered especially challenging as these animals have a relative small stomach and rapid gut passage [16]. In the previously reported moose case [8], yew remnants could only be detected following thorough study of the rumen content. In some studies analytic detection of taxines within the stomach content or tissue samples have been used to support the diagnosis [14], [17].

The identification of yew intoxication in a moose in Norway in 2008 [8] led to an increased awareness in our laboratory and subsequent diagnosis of further cases in moose and roe deer. Yew intoxication in reindeer (*Rangifer tarandus tarandus*) has also been diagnosed. This study summarizes these intoxication cases and describes an assay used to verify the presence of taxines in moose tissue samples.

## Materials and methods

### Animals and background

The material comprised five moose and seven roe deer from south-eastern Norway and two reindeer from northern Norway. The moose were recovered during the winter months (December-February) between 2009 and 2011 in the municipalities of Røyken 59°43'N, 10°27'E; Nannestad 60°16'N, 10°57'E; Aurskog-Høland 59°50'N, 11°31'E and Sande 59°36'N, 10°15'E. Six of the roe deer were found in Oslo 59°54'N, 10°45'E during the winters 2015 (five animals) and 2016 whereas the seventh roe deer was detected in the municipality of Rygge 59°22'N, 10°40'E in January 2016. The reindeer belonged to a semi-domesticated herd situated on the island of Kvaløya 69°62'N, 18°51'E, Tromsø municipality and were found during the winters of 2001 and 2007.

**Fig 1:**
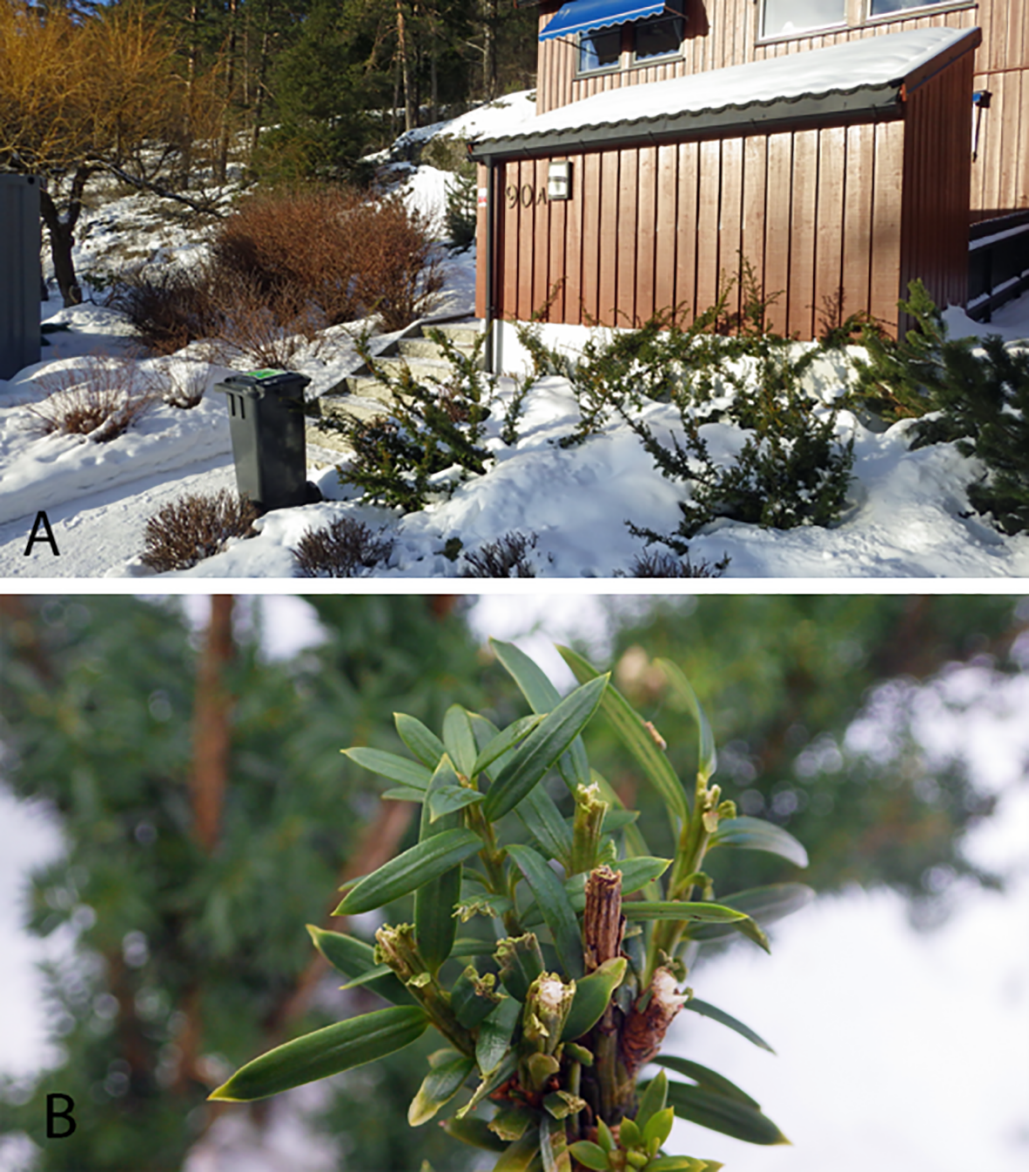
The housing estate in Oslo where one yew-intoxicated roe deer was found dead. (**A**) Terraced house with open access from woodland and deer hoof imprints in the snow in front of a yew hedge. (**B**) Browsing damage on one of the yew plants.

Two of the roe deer were found in a moribund state and subsequently euthanized due to animal welfare reasons. The culling was performed by a shot against the brain and was carried out by authorized personal from the Local Wildlife Authority. All other animals were found dead. Most animals were detected in close proximity to, or within private gardens and they appeared to have collapsed without struggle. In gardens which were inspected there was typically free access from woodland and the presence of yew cultivars showing browsing damage (Fig 1A and 1B). The moose cases included calves (< 1 year) and adults of both sexes. The roe deer consisted of one adult female and twin kids found together, three further adults (two females, one male) and one kid. The reindeer comprised one adult female and one calf.

### Pathology, bacteriology and tissue sampling for chemical analysis

All carcasses were autopsied in the laboratory following standard procedures. The rumen was inspected for the presence of yew plant tissues and if not readily detected, as was the case in one moose, a total of 1 kilogram of the rumen content was collected and later carefully examined under optimal light conditions. Samples for bacteriological culture were routinely taken from the lungs and liver, and occasionally the spleen. The samples were sown onto bovine blood agar plates that were incubated aerobically and anaerobically for two days at 37°C. Samples of the brain, lungs, heart, liver, kidneys and skeletal muscles were fixed in 10% buffered formalin, embedded in paraffin, sectioned at 5μm and stained with haematoxylin and eosin for histopathological examination.

Fresh tissue samples from the heart and liver were obtained from the five moose cases and stored frozen at -20°C until taxine analysis. Samples from two moose autopsied during the winter with the diagnoses of cachexia and trauma respectively were included as negative controls.

### Alkaloid extraction and analysis of *Taxus* leaves and tissue samples

Since taxine standards are not commercially available and the production of in-house calibrants was not possible within the framework of the current project, the objective of the chemical analyses was to qualitatively show the presence of major yew alkaloids in liver and heart samples. In order to achieve this, a crude alkaloid extract from yew leaves was analyzed and used as a reference. The same approach has previously been applied for the diagnosis of yew intoxication in humans [18].

Two branches of an ordinary garden *Taxus* sp were harvested during winter in Skytta 59°59'N, 10°54'E, Nittedal municipality, south-eastern Norway. One gram of leaves was removed and extracted with 10 mL of methanol/water (9:1, v/v) by orbital shaking (175 min^-1^) for 30 min. An aliquot of the extract (1 mL) was transferred into a glass tube and evaporated to dryness under a stream of nitrogen (40°C). The residue was dissolved in 5 mL 1 M HCl by vortexing. The solution was transferred into a separatory funnel and washed with 5 mL of dichloromethane and the organic phase discarded. The aqueous phase was then alkalized to pH 9 with ammonia solution (25%) and extracted 3 times with 5 mL dichloromethane. The combined organic phases were evaporated using rotary evaporation resulting in a pale yellow, oily residue, comprising a crude alkaloid mixture. The mixture was dissolved in 5 mL acetonitrile/water (1:1), and aliquots were filtered through a 0.22 μm nylon membrane (Costar, Corning Inc., Corning, NY) and subjected to high performance liquid chromatography–ion trap mass spectrometry (LC-ITMS) analysis.

The sample preparation procedure for the moose heart and liver samples was based on a previously published method, with modifications [18], [19]. The tissue samples were allowed to thaw before portions of 0.5 g were cut and homogenized (Ultra-Turrax T25; IKA-Werke GmbH & Co. KG, Staufen i. Br., Germany) in 2 mL 10 mM ammonium carbonate for 2 min. Homogenates were centrifuged and the supernatants transferred to Oasis HLB SPE columns (60 mg; Waters, Milford, MA), which were preconditioned with 1mL methanol, 1 mL water and 1 mL 10 mM ammonium carbonate. The sample extracts were passed through the columns with constant flow. The cartridges were washed with 3 mL 10 mM ammonium carbonate and dried for 1 min. The columns were then eluted with 1 mL methanol, which was evaporated to dryness under a stream of nitrogen (40°C). Individual residues were dissolved in 250 μL acetonitrile/water (1:1) using sonication (10 min). The solutions were filtered through a 0.22 μm nylon membrane (Costar) and subjected to LC-ITMS analysis.

The mass spectrometer used was a LTQ linear ion trap mass spectrometer, with an electrospray ionization interface operated in the positive ionization mode (Thermo Fisher Scientific, Waltham, MA). Chromatography was performed using a 100 × 2.0 mm i.d., 4 μm, Synergi-Fusion RP column (Phenomenex, Torrance, CA) using a mobile phase consisting of an aqueous solution of 2.5 mM ammonium carbonate (A) and acetonitrile (B) at 0.3 mL/min. The column was isocratically eluted using a mobile phase consisting of 82:18 A/B for 0.5 min, and then by linear gradient elution to 38% B over 11.5 min, and then to 95% B over 3 min. The column was flushed with 95% B for 2 min, before returning to the starting conditions.

The mass spectrometer was run in full-scan mode in the mass range *m/z* 300–1000. Simultaneous collision-induced fragmentation of the three most intense ions was achieved using data-dependent scanning (intensity threshold level of 10^4^, isolation width 2 *m/z*, activation Q set to 0.25, activation time 30 ms). A separate MS method was set up for targeted fragmentation of the protonated molecular ions of putative major taxines in the alkaloid extract and tissue extracts. Taxines were tentatively identified by their molecular mass and the presence of the principal fragment ion in their MS^2^ spectra resulting from cleavage of the ester linkage between the diterpene and dimethylamino-phenylpropionic acid part of the molecules (*m/z* 194 for taxine B-derived analogues and *m/z* 210 for taxine A-derived analogues). The electrospray interface was operated with a source voltage of 5 kV, capillary temperature of 250°C, capillary voltage of 47 V, tube lens offset of 130 V, sheath gas flow of 48 units (approximately 48 L/min) and an auxiliary gas flow of 20 units (approximately 20 L/min). For all MS fragmentation experiments the relative collision energy was 35%.

## Results

### Pathological and bacteriological findings

**Fig 2:**
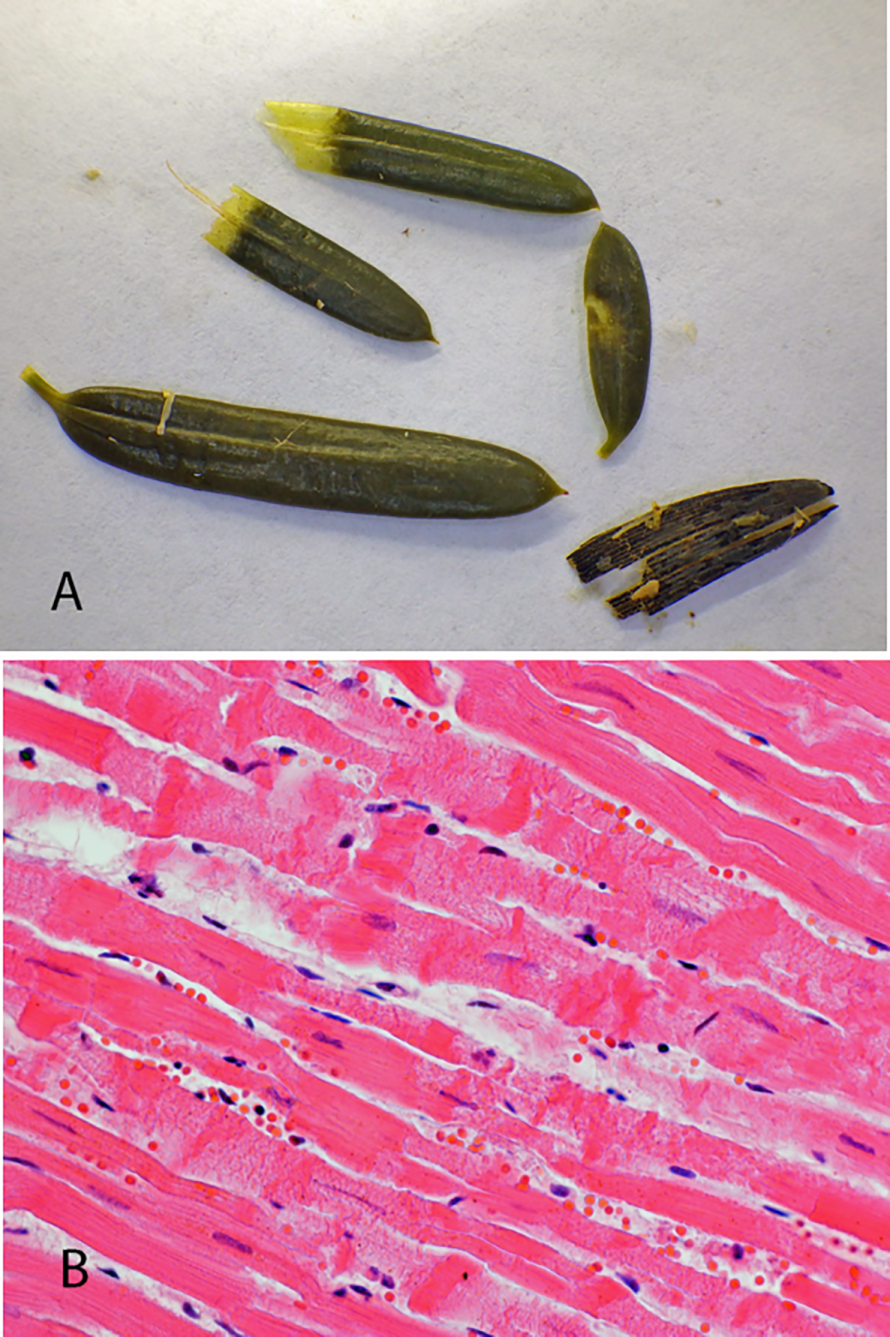
Pathological findings in a yew-intoxicated roe deer. (**A**) Yew leaves from the rumen. (**B**) Acute lesions in the myocardium with hemorrhage, swelling and segmental fragmentation of muscle fibers, loss of cross striation and granular appearance, and hypercontracted sarcomers (contraction bands).

All carcasses were in normal condition and decomposition varied from moderate to considerable. No pathogenic bacteria were isolated from the internal organs. Common gross pathological findings included congestion in the chest aperture and lungs, lung edema, bilateral heart dilatation and moderate amounts of serous fluid in the thoracic cavity and pericardial sac. Some of the animals also showed petechial to ecchymotic hemorrhage in the epi- and endocardium and two of the moose displayed greatly enlarged (congested) spleens. Moderate to considerable quantities of yew leaves and branches were readily recognizable in the rumen content of all animals (Fig 2A) with the exception of a single moose. In this moose, yew remnants could not be detected by detailed examination of the rumen content. In one reindeer, yew tissues were found alongside *Rhododendron* sp. tissues.

Histopathological evaluation of internal organs was often complicated by autolysis, particularly in moose. Histological findings included general congestion and lung edema in most animals, and the presence of myocardial lesions in the roe deer. These myocardial lesions included multifocal acute myocyte degeneration and necrosis with hemorrhage (Fig 2B), occasionally accompanied by minute inflammatory cell infiltration.

### Chemical analyses

**Fig 3:**
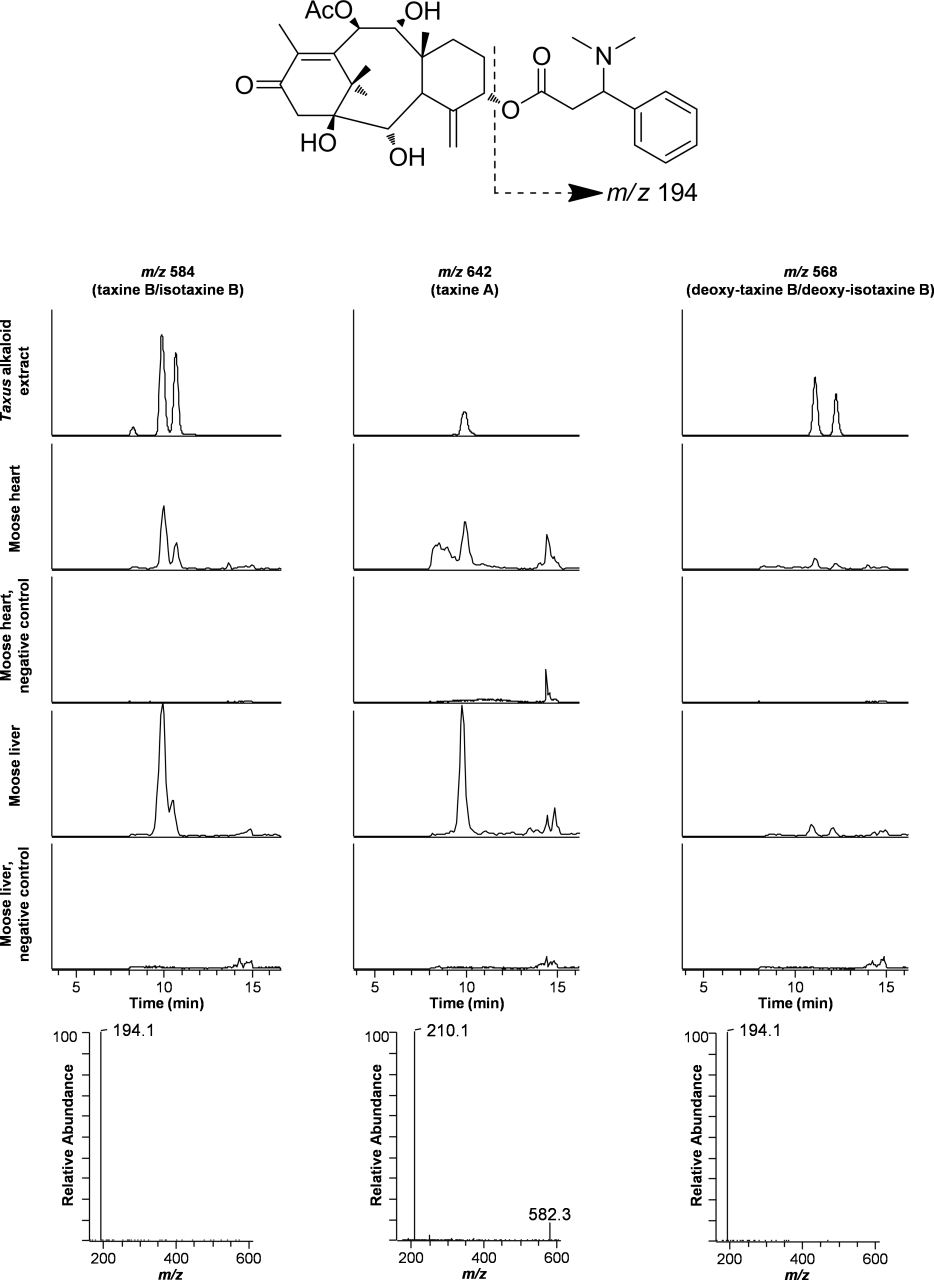
*Taxus* alkaloids (taxines) in yew leaves and moose tissue samples. Extracted ion chromatograms from liquid chromatography–ion trap mass spectrometry analysis of an alkaloid extract of yew leaves (*Taxus* sp.), and heart and liver samples from a yew-intoxicated moose and a negative moose control. Individual traces of taxine B/isotaxine B, taxine A and deoxy-taxine B/deoxy-isotaxine B are shown on a fixed scale in order to visualise relative concentration differences (alkaloid extract 1.5 × 10^6^; tissue samples 4.0 × 10^4^). MS^2^ spectra (below) were obtained from fragmentation of the protonated molecular ions of corresponding taxines in the yew extract. The principal MS^2^ fragment is due to cleavage of the dimethylamino-phenylpropionic acid part of the molecules as shown for taxine B above.

The taxines found in the crude alkaloid extracts from yew leaves appeared to be a complex mixture of different congeners, but the precise chemical diversity was not studied in detail within the present project. According to the literature, 11 different taxines have been described, with taxine B, which isomerizes easily into isotaxin B, the common major alkaloid detected [9], [20]. Two compounds within the alkaloid extract affording ions of *m/z* 584 in the LC-MS chromatograms were thus tentatively identified as the protonated molecular ions of taxine B and isotaxine B (Fig 3). The identity of these compounds was further verified by LC-ITMS^2^, where the mass analyzer was set to fragment *m/z* 584. The product ion spectra of the two compounds were dominated by a fragment ion with *m/z* 194, which corresponds to protonated 3-dimethylamino-3-phenylpropionic acid [19] (Fig 3). Other prominent taxines included two compounds of 16 mass units less than taxine B and isotaxine B, but with similar product ion spectra (Fig 3). These compounds were likely deoxy-analogues of taxine B, which have been previously reported in the literature [9]. In contrast to taxine B, the alkaloid part of taxine A is hydroxylated, and the diterpene part of the molecule is acetylated resulting in a molecular weight of 641 Da. Thus, the protonated molecular ion of taxine A was expected at *m/z* 642. A corresponding compound was found in the LC-ITMS chromatograms at a similar retention time as taxine B/isotaxine B. The principal product ion in the MS^2^ spectrum of *m/z* 642 was observed at *m/z* 210, which is equivalent with hydroxylation of the dimethylamino-phenylpropionic acid part in taxine A (Fig 3). These five congeners represented the major taxine congeners in the yew alkaloid extract.

All five major taxines found in the crude yew leave extracts were detected with good signal/noise ratio in the extracts of heart and liver from the four intoxicated moose with visible yew remnants in their rumen (Fig 3). The observed LC-ITMS peaks believed to correspond to individual taxines in these tissue samples were strong enough to allow acquisition of product ion spectra. Thus, the product ion spectra following fragmentation of the protonated molecular ions of assumed taxines in the tissue samples were identical with those of the major taxines in the yew alkaloid extract. The relative peak areas of individual taxines were generally 2–6 times larger in liver extracts compared to the heart. In the moose with no visible yew rumen content, the same major taxines were detected, but the signal/noise of the corresponding LC-ITMS peaks was relatively low. Taxines were not detected in the heart and liver tissues of the two control animals.

## Discussion

This is the first report of yew intoxication in roe deer and reindeer. We also document additional cases of the same intoxication in moose, previously reported in a single animal [8]. The intoxicated animals were in a normal winter body condition and were found dead with no signs of struggle. Post mortem examination revealed the presence of yew remnants within the rumen and non-specific gross lesions pointing to acute circulatory (cardiac) collapse. One of the reindeer had browsed the cardiotoxic plant *Rhododendron* [21] along with yew and presumably died from a combined effect of the two toxic plants. In the moose cases, the diagnosis was supported by the demonstration of major yew taxines in heart and liver samples by LC-ITMS. The four moose with grossly visible yew remnants within the rumen and strong taxine tissue signals, presumably died from acute intoxication whereas the fifth moose, with no detectable yew within the rumen and weaker taxine tissue signals can be suggested to have died after a sub-acute course of intoxication. The latter finding demonstrates that it is imperative to consider *Taxus* poisoning when evaluating the cause of sudden death in wild ruminants, even if there are no grossly identifiable yew plant tissues within the rumen. We have also demonstrated yew remnants in the rumen of poorly nourished moose and roe deer found dead during winter. However, due to the emaciated body condition of these individuals it could not be concluded whether they died from starvation or intoxication.

Due to the normally rapid course of yew intoxication, with livestock dying acutely within a few hours after exposure, definitive gross lesions are often absent and histological lesions are rarely reported [9], [10]. Gross lesions reported include pulmonary edema, pericardial effusion, splenic congestion and cardiac hemorrhage [11], [13], [15], [22]. All of these lesions were seen in the intoxicated cervids in the present study. Histological lesions, in the form of multifocal myocardial degeneration and necrosis as seen in the roe deer has previously been recognized only in single cases of intoxication in livestock [15], [22]. Taxines cause an increase in cytosolic calcium [9] which is detrimental to cells in general. It has been suggested that myocardial lesions related to yew intoxication are a result of toxic accumulation of calcium in cardiac myocytes [14], and that the development of these lesions is dependent on the duration of disease [22]. This is supported by a study reporting the absence of cardiac lesions in cattle dying acutely after yew exposure whereas significant myocardial degeneration and necrosis occurred in an animal that died several days after exposure [15]. Further progression of these lesions to extensive myocardial fibrosis has been reported in a bovine calf that died 18 days following yew exposure [14]. The finding of cardiac lesions in roe deer but not in moose and reindeer in the present study might indicate a less acute course of intoxication in the roe deer compared to the two other species. However, it must be emphasized that these apparent differences might also be artificial due to variable degrees of tissue autolysis camouflaging subtle myocardial lesions.

Species differences in taxine tolerance are well documented for domestic animals [9] and are likely to occur also among deer species. The pharmacokinetics of taxines has not been investigated but their main metabolization is likely to be by P450 enzymes within hepatocyte microsomes, as demonstrated for the related alkaloids paclitaxel and docetaxel used as anti-neoplastic drugs in the human medicine [10]. In ruminants, microbiological taxine degradation in the rumen is likely to be involved in the detoxification process as demonstrated for white-tailed deer [7]. However, a life-time adapted tolerance through induction of detoxifying liver enzymes can be suggested to be the key factor underlying the apparent protection against yew taxines observed in cervids such as roe deer and lethal yew poisoning can be considered to be dependent on the quantity consumed at first intake [8]. Regarding adaptation, the roe deer may benefit from its feeding behavior that includes a diet composed of small amounts of a wide range of plants. This browsing behavior can be considered beneficial by reducing the relative toxic dose and thus risk of acute intoxication after the first exposure to yew.

Yew is rare on a national scale in Norway but occurs in coastal and lowland areas in southern Norway [23]. Cultivars of yew are, however, widely used evergreen plants in gardens and public areas all over the country, and ornamental yew plants were most likely the main source of intoxication in our study. Horticultural yew plants obviously represent a potential threat to wild cervids during the winter when food is scarce and covered by snow, especially when located close to woodland areas. All but two of the reported roe deer cases occurred in Oslo in January-February 2015. This particular winter, heavy snow cover and icing limited the access to the blueberry plant, a key plant in the winter diet of roe deer.

We propose that yew intoxication is underestimated as a cause of death in cervids, particularly roe deer. Roe deer are small and their carcasses are not readily observed. Individuals browsing on garden yew plants over a short nocturnal period and returning to the woods before dying will easily be overlooked and eaten by scavengers. During the media focus on the roe deer cases in Oslo in 2015, we received a report of a well-monitored and supplementary fed rural flock of approximately 10 individuals that was reduced by half following a single nights browsing on a nearby yew planting. Although no carcasses were detected, we suggest that this sudden flock reduction may have occurred as a result of yew intoxication.

In conclusion, this study demonstrated that the yew plant represents a potential intoxication hazard for various cervids. The planting of ornamental yew should be avoided in locations close to larger woodland areas. Our findings challenge previous assumptions of a generally high tolerance to yew taxines in cervid species such as roe deer. Adaptation through induction of protective mechanisms in the liver following a sub-lethal primary exposure presumably plays a key role for the outcome after subsequent yew ingestion. A follow up of the present study would be to examine taxine degradation in freshly harvested hepatocyte microsomes from Norwegian cervid populations with and without presumed exposure to yew. This could highlight both species differences in taxine tolerance and the role of individual adaptation.
